# The Influence of *β*-Cyclodextrin on the Reduction of Cholesterol Content in Egg and Duck Liver Pâté

**DOI:** 10.3390/foods8070241

**Published:** 2019-07-03

**Authors:** Leocadio Alonso, María V. Calvo, Javier Fontecha

**Affiliations:** 1 Instituto de Productos Lácteos de Asturias (CSIC), 33300 Asturias, Spain; 2 Instituto de Investigación en Ciencias de la Alimentación (CSIC-UAM), 28049 Madrid, Spain

**Keywords:** *β*-cyclodextrin, cholesterol, fatty acids, egg, duck liver pâté

## Abstract

The use of *β*-cyclodextrin (*β*-CD) to reduce cholesterol is increasing in food research due to its affinity for non-polar molecules such as cholesterol. The objective of this study was to study the ability of *β*-CD to remove cholesterol in natural egg, powdered egg, and duck liver pâté and its effect on individual fatty acids. A concentration of 5% of *β*-CD was found to be a suitable amount to remove 80.04 ± 4.96–82.12 ± 5.36% of cholesterol from yolk and powdered eggs and 80.21 ± 5.28% of cholesterol from duck liver pâté. *β*-CD complexed to cholesterol was precipitated and removed by centrifugation. Individual fatty acid compositions did not differ (*p* < 0.05) between the controls and the products treated with 5% *β*-CD.

## 1. Introduction

Consumers have been reducing their fat intake in order to reduce their intake of cholesterol and therefore lower their risk of coronary heart disease [1]. This trend has affected a number of foodstuffs that are considered to increase the risk of cardiovascular diseases because they contain a high proportion of cholesterol. Methods for reducing cholesterol in foods have been developed, including blending the foods with vegetable oils [2,3], extraction of cholesterol by distillation and crystallization [4,5], adsorption of cholesterol with saponin and digitonin [6,7], assimilation of cholesterol by enzymes from microorganisms [8,9], and removal of cholesterol by supercritical carbon dioxide extraction [10,11]. In recent years, studies have been published that describe the use of *β*-cyclodextrin (*β*-CD) in foods [12,13,14]. *β*-CD can be used to remove cholesterol from milk and dairy products and lard [3,15,16,17]. *β*-CD is a cyclic oligosaccharide consisting of seven glucose units. The molecule of *β*-CD is doughnut shaped and its central portion is a circular hydrophobic space similar in diameter to a cholesterol molecule, giving the molecule an affinity for non-polar molecules such as cholesterol, and providing the highly specific ability of *β*-CD to form an inclusion insoluble complex [18,19].

The objective of this work was to determine the use and influence of *β*-CD on natural egg yolk, powdered egg yolk, and duck liver pâté for manufacturing low cholesterol food products.

## 2. Materials and Methods

### 2.1. Samples and Reagents

Natural eggs were purchased from the local market, and the yolk was separated from the albumen manually. Powdered egg yolk was supplied by the company Huevos Maryper (Murcia, Spain) and duck liver pâté by Malvasia, S.A (Soria, Spain).

*β*-CD (purity 99.5%) was purchased from Shandong Xinda Fine Chemical Co., Ltd. (Qingdao, China). Standards of fatty acid methyl esters, cholesterol, cholestane, and all reagent grades were supplied from Sigma (St. Louis, MO, USA).

### 2.2. Cholesterol Removal

Egg yolk and duck liver pâté dissolved in deionized water in a ratio of 1:2 (wt/vol) were treated with 1, 3, 5 and 7% of *β*-CD (mass 1134.987 g/mol) by the method described by Alonso et al. [14] with some modifications. 100 g of samples containing 5% of *β*-CD (wt/wt) were placed at room temperature and were stirred at 200 rpm for 60 min. After mixing, the treated samples were left standing overnight at 4 °C (to allow time to encapsulate cholesterol to *β*-CD and to precipitate the cholesterol–*β*-CD complex at the bottom of the beaker). The upper layer (supernatant) without the cholesterol–*β*-CD complex was separated by centrifugation at 3000 rpm for 10 min, and the amount of cholesterol was determined. The sediment containing the inclusion complex (cholesterol–*β*-CD) was also analyzed for cholesterol.

### 2.3. Lipid Extraction

Lipids were extracted from samples following a procedure described by an International Standard [20].

### 2.4. Determination of Cholesterol

The technique chosen to determine the amount of cholesterol was as described by Alonso et al. [21].

### 2.5. Fatty Acid Analysis

Fatty acid methyl esters (FAME) were prepared by alkaline catalyzed methanolysis of the extracted lipids using the method described by Alonso et al. [22].

### 2.6. Statistical Analysis

All treatments were made in triplicate, and three analyses for each experiment were done. Experimental data were treated by analysis of variance (ANOVA) using the statistical software SAS (version 8.02, SAS Institute Inc., Cary, NC, USA). Mean values for the different treatments were compared using the Bonferroni test (SPSS software 15.0; Chicago, IL, USA). Differences (*p* > 0.05) were considered statistically significant.

## 3. Results and Discussion

**Figure 1:**
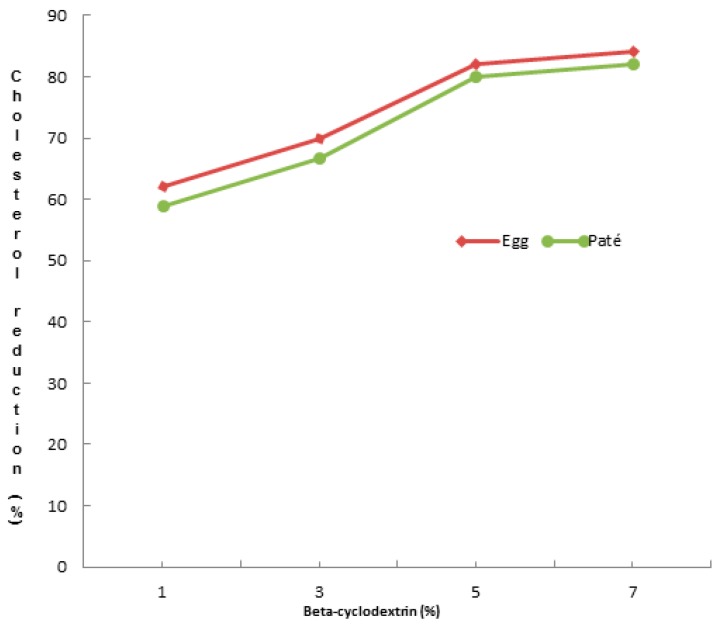
Effect of *β*-cyclodextrin (*β*-CD) concentrations (1, 3, 5 and 7%) on the removal of cholesterol from egg yolk and duck liver pâté.

**Figure 2:**
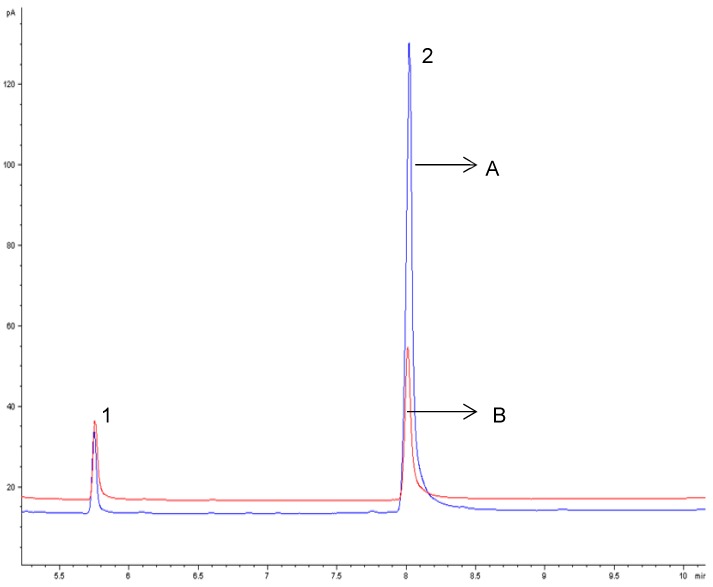
Gas chromatographic profiles of cholesterol in egg yolk from the control (A) and egg yolk treated with *β*-CD (B). Peak identification: 1 = cholestane, 2 = cholesterol.

**Figure 3:**
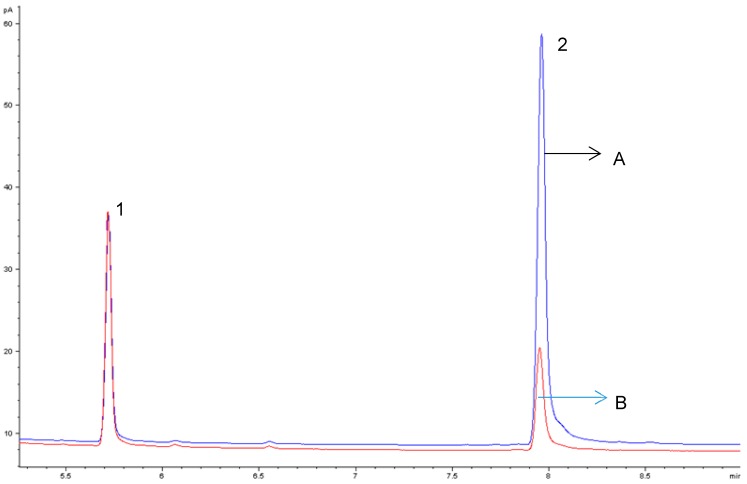
Gas chromatographic profiles of cholesterol in duck liver pâté from the control (A) and duck liver pâté treated with *β*-CD (B). Peak identification: 1 = cholestane, 2 = cholesterol.

**Figure 4:**
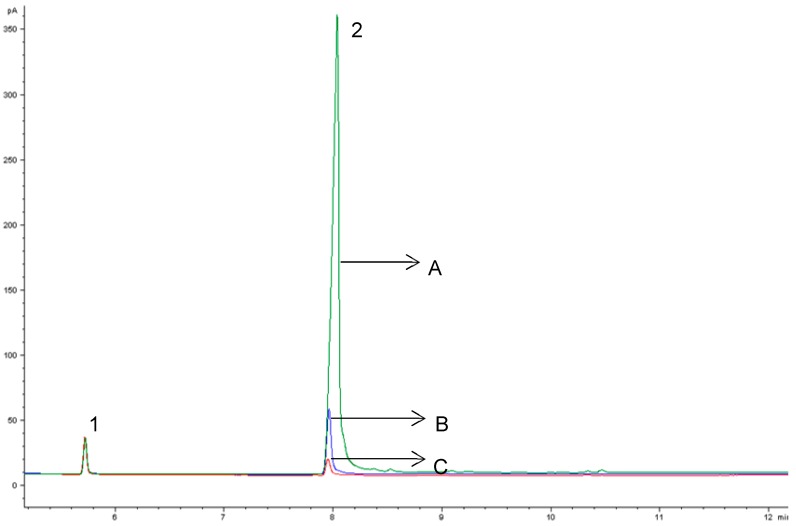
Comparison of gas chromatographic profiles of cholesterol in duck liver pâté from the control (A) and duck liver pâté treated with *β*-CD (B) and the precipitated *β*-CD–cholesterol complex separated by centrifugation (C). Peak identification: 1 = cholestane, 2 = cholesterol.

Different concentrations of *β*-CD (1, 3, 5, and 7%) were assayed into egg yolk (natural and powdered) and duck liver pâté, to optimize the removal of cholesterol. The concentrations of *β*-CD removed 62.12 ± 5.07% to 82.15 ± 5.36% of cholesterol when mixed at room temperature for 60 min at 200 rpm (Figure 1). There were no differences (*p* < 0.05) between 5% and 7% of *β*-CD on cholesterol removal, and we chose the concentration of 5% of *β*-CD as a suitable amount for removing cholesterol from natural and powdered egg yolk and duck liver pâté (Table 1). Cholesterol reduction in natural and powdered egg yolk (Figure 2) ranged from 80.04 ± 4.96% to 82.12 ± 5.36% and 80.21 ± 5.28% in duck liver pâté (Figure 3) compared to the controls. These results for the reduction of cholesterol in egg are similar to those found by Borges et al. [23] in a study of optimizing the extraction of cholesterol from dehydrated egg yolk using acetone that resulted in an 81% reduction. The use of arabic gum reported by Villarreal et al. [24] allowed between 83.85% and 93.26% of cholesterol to be removed in egg yolk. These values are higher than those found in our study, but arabic gum has properties of anionic polysaccharides, which could form insoluble electrostatic complexes with proteins. Another anionic polysaccharide used as a chelating agent [25] was mesquite gum, which extracted 80.31–87.75% of cholesterol in egg yolk. Similar results were found by Jeong et al. [26] (around 80% reduction of cholesterol) in a study of cholesterol removal from whole egg using a crosslinked *β*-CD. Figure 4 shows a chromatogram of cholesterol analysis of duck liver pâté treated with *β*-CD and the sediment complex with the encapsulated cholesterol. The *β*-CD–cholesterol complex is eliminated in the separation steps of the cholesterol reduction process by centrifugation during the separation process.

Table 2 shows the mean values of fatty acids (%) from the fat from controls and from natural egg yolk, powdered egg yolk, and duck liver pâté treated with 5% of *β*-CD. The concentration of individual fatty acids did not exhibit significant differences (*p* < 0.05) between the fat from the controls and the products treated with *β*-CD. No differences (*p* < 0.05) were found for total saturated fatty acids (SFA) (C14:0 + C16:0 + C18:0) from the controls and the natural egg, powdered egg, and liver duck pâté treated with 5% of *β*-CD. The same pattern were also observed for the average content of total unsaturated fatty acids (UFA) (C16:1 + C18:1 + C18:2 + C18:3) before and after treatment with 5% of *β*-CD, which did not show any differences (*p* < 0.05) for natural egg yolk, powdered egg yolk, or duck liver pâté. There is no information in the literature showing the effect of the treatment of egg yolk or duck liver pâté with *β*-CD on fatty acid composition, and this is the first time that this has been shown. Chen et al. [27] and Gonzalez et al. [10] using supercritical fluid extraction observed that milk fat had differences in fatty acid composition compared to the control. Similar results to our study were found by Alonso et al. [28] in a study of the effect of *β*-CD on the fatty acids of milk fat, suggesting that treatment with *β*-CD did not affect fatty acid composition.

## 4. Conclusions

The results of this study suggest that treatment with *β*-CD can be used on egg (natural and powdered) and liver duck pâté to make low-cholesterol foodstuffs without altering the fatty acid composition. As a result, the present study can be used in the development of low-cholesterol functional foods for people with hypercholesterolemia.

## Figures and Tables

**Table 1:** Cholesterol (mg cholesterol/10 g fat) content and reduction of cholesterol (%) in natural egg yolk, powdered egg yolk, and duck liver pâté from the control and those treated with 5% of *β*-cyclodextrin (*β*-CD).

Sample	Control	5%-*β*-CD	Reduction (%)
Natural egg yolk	396.54 ± 28.94	71.37 ± 5.45	82.12 ± 5.36 *
Powdered egg yolk	383.59 ± 25.64	76.72 ± 5.96	80.04 ± 4.91 *
Duck liver paté	78.64 ± 6.85	15.73 ± 0.12	80.21± 5.28 *

Values are means ± standard deviation of triplicate analyses. * significance (*p* > 0.05). *n* = 9.

**Table 2:** Fatty acid (%) composition of natural egg yolk, powdered egg yolk, and duck liver pâté from the control and those treated with 5% *β*-cyclodextrin (*β*-CD).

Fatty Acid	Natural Egg Yolk		Powdered Egg Yolk		Duck Liver Paté	
	Control	5%-*β*-CD	Control	5%-*β*-CD	Control	5%-*β*-CD
C14:0	0.21 ± 0.03	0.23 ± 0.03	0.29 ± 0.04	0.27 ± 0.04	0.38 ± 0.05	0.41 ± 0.05
C16:0	24.63 ± 1.35	24.22 ± 1.48	23.47 ± 1.42	23.50 ± 1.52	32.40 ± 1.68	32.97 ± 1.72
C16:1	3.25 ± 0.12	3.27 ± 0.15	2.75 ± 0.13	2.63 ± 0.15	0.60 ± 0.18	0.57 ± 0.16
C18:0	8.41 ± 0.68	8.45 ± 0.62	7.72 ± 0.58	7.42 ± 0.51	26.94 ± 1.95	27.40 ± 2.02
C18:1	46.37 ± 1.96	45.68 ± 2.01	49.02 ± 2.12	49.53 ± 2.24	22.21 ± 1.52	20.68 ± 1.69
C18:2	12.86 ± 1.10	13.84 ± 1.21	12.19 ± 1.15	12.38 ± 1.19	16.92 ± 1.18	17.36 ± 1.22
C18:3	4.26 ± 0.41	4.38 ± 0.39	4.07 ± 0.34	4.05 ± 0.31	0.56 ± 0.04	0.61 ± 0.04
Total SFA	33.54 ± 1.61	32.90 ± 1.72	31.48 ± 1.65	31.19 ± 1.73	9.72 ± 1.79	60.78 ± 2.44
Total UFA	66.74 ± 3.28	67.17 ± 3.47	68.05 ± 3.49	68.59 ± 3.66	40.29 ± 2.89	39.22 ± 3.08

SFA: saturated fatty acid; UFA: unsaturated fatty acid; Values are means ± standard deviation (g fatty acids/100 g of fatty acids) of triplicate analyses. *n* = 9. There were no significant differences between the means of the controls and the *β*-CD samples for any of the fatty acids (*p* > 0.05).
